# Enhanced ZNF521 expression induces an aggressive phenotype in human ovarian carcinoma cell lines

**DOI:** 10.1371/journal.pone.0274785

**Published:** 2022-10-03

**Authors:** Stefania Scicchitano, Ylenia Montalcini, Valeria Lucchino, Valentina Melocchi, Valerio Gigantino, Emanuela Chiarella, Fabrizio Bianchi, Alessandro Weisz, Maria Mesuraca

**Affiliations:** 1 Laboratory of Molecular Haematopoiesis and Stem Cell Biology, Department of Experimental and Clinical Medicine, University Magna Græcia, Catanzaro, Italy; 2 Laboratory of Stem Cell Biology Department of Experimental and Clinical Medicine University Magna Graecia, Catanzaro, Italy; 3 Unit of Cancer Biomarkers, Fondazione IRCCS–Casa Sollievo Della Sofferenza, San Giovanni Rotondo (FG), Italy; 4 Laboratory of Molecular Medicine and Genomics, Department of Medicine, Surgery and Dentistry, University of Salerno, Baronissi (SA), Italy; 5 Genome Research Center for Health, University of Salerno Campus, Baronissi (SA), Italy; Virginia Commonwealth University, UNITED STATES

## Abstract

Epithelial ovarian carcinoma (EOC) is the most lethal gynecological tumor, that almost inevitably relapses and develops chemo-resistance. A better understanding of molecular events underlying the biological behavior of this tumor, as well as identification of new biomarkers and therapeutic targets are the prerequisite to improve its clinical management. ZNF521 gene amplifications are present in >6% of OCs and its overexpression is associated with poor prognosis, suggesting that it may play an important role in OC. Increased ZNF521 expression resulted in an enhancement of OC HeyA8 and ES-2 cell growth and motility. Analysis of RNA isolated from transduced cells by RNA-Seq and qRT-PCR revealed that several genes involved in growth, proliferation, migration and tumor invasiveness are differentially expressed following increased ZNF521 expression. The data illustrate a novel biological role of ZNF521 in OC that, thanks to the early and easy detection by RNA-Seq, can be used as biomarker for identification and treatment of OC patients.

## Introduction

Epithelial ovarian carcinoma is the leading cause of gynecological cancer-related mortality, with 295,000 new cases and 184,000 deaths worldwide in 2020 [1]. Due to the lack of effective screening programs two-thirds of patients with OC are diagnosed with advanced disease. Despite a generally good response to surgery and first-line chemotherapy, OC almost inevitably relapses in different peritoneal sites and develops chemo-resistance. Only 45% of OC-affected women are likely to survive for five years, compared to 89% of breast cancer patients [2,3]. Despite increased radicality of debulking surgery and modifications of conventional chemotherapies there have been only modest gains for overall survival (OS) in OC in the last decades [4]. Based on their histological and morphological classification OC can be divided in serous, mucinous, endometrial, clear cells and transitional cells (depending on the tissue of origin involved). Moreover, OCs can be divided in type I (low-grade tumor with BRAF and KRAS mutations) or type II (high-grade tumor with a variety of mutations including HOX, PTEN, KRAS, AKT1, BRCA1/2 genes) [5–10] and various dysregulated mechanisms underlying the pathology [11–13]. An important therapeutic target in OC may be represented by a subpopulation of cells defined as ovarian cancer stem cells (OCSCs) [14] and considered a key factor in cancer initiation, relapse and resistance to therapy [15,16]. The stem cell-associated transcription co-factor, zinc finger protein 521 (ZNF521) is a transcription factor abundant in OC, where a high expression is associated with poor prognosis [17,18]. ZNF521 has been recently included in a list of top 15 genes associated to poor survival in serous cystadenocarcinomas [19]. A significant number of gene amplifications for ZNF521 are detected in ovarian cancers (~6%) [20]. This association with oncogenesis with a role in control of HSCs (Hematopoietic Stem Cells) [21], LSCs (Leukemic Stem Cells) [22–25] and in iNSCs (immortalized Neural Stem Cells) [26–28] as well as in mesenchymal differentiation [29–34], involves ZNF521 in the control of the Stem Cells (SCs) compartment and in Cancer Stem Cells (CSCs). ZNF521 has also been implicated in miRNA regulation [35] where miR-9, via targeting Zfp521 (murine ortholog to human ZNF521), could promote the neural differentiation of mouse bone marrow mesenchymal stem cells (MSCs) [36]. It is notable that breast and ovarian cancers with BRCA1 mutations are inhibited by miR-9 [37]. Regulation of ZNF521 was also found in gastric cancer cells by miRNA-204-5p [38] and in hepatocellular carcinoma by miR-802 [39]. Additionally, in ovarian cancer an integration analysis of microRNA and mRNA gave a positive association of ZNF521 with the miR34-family and miR133b [18].

As the mortality rate of this tumor has improved only marginally over the past decades, a better understanding of the molecular events that drive its development, maintenance and progression, and the identification of new biomarkers and therapeutic targets, are paramount to improve OC treatment and hence its prognosis. In this study the role of ZNF521 is investigated in the regulation of human ovarian carcinoma cells.

## Materials and methods

### Cell lines and culture conditions

The differentiated papillary human ovary cystoadenocarcinoma cell line HeyA8, the human desmoplastic cerebellar medulloblastoma DAOY cells and the human embryonic kidney HEK293T cells were cultured in DMEM. The human ovarian adenocarcinoma cell line ES-2, the acute monocytic leukemia THP1 cells and the lymphoblastoid B (multiple myeloma) IM9 cells were cultured in RPMI. Cell culture medium are supplemented with 10% fetal bovine serum, 50U of penicillin and 50μg of streptomycin/ml (Thermo Fisher Scientific, Milan, Italy) and cell lines were maintained at 37°C in 5% CO_2_.

### Transfection and transduction of cell lines

Lentiviral production was carried out in 100 mm tissue culture plates, where the cell line HEK293T were transfected with 10μg of plasmid for FUIGW (control vector) or FUIGW-ZNF521 [40] plus lentiviral packaging plasmids (2μg of pCMV-VSVG and 10μg of pCMV-deltaR8-91), using the calcium phosphate method. Lentiviral media were collected at 24, 48 and 72 hours after transfection and supplemented with 6μg/ml of polybrene [41] and ovarian carcinoma cell lines HeyA8 and ES-2 were stably transduced with the control vector FUIGW or FUIGW-ZNF521. Three rounds of transduction were performed by centrifuging the cells with lentivirus at 3200 rpm at 32°C for 50min. Cells were 70–80% positive for the transgene EGFP by FACS analysis and stably expressed the ZNF521 protein. The transduction was performed in three independent experiments and cells were further sorted for EGFP giving a homogeneous population over 90% positive.

### Proteins extracts and Western blotting

HeyA8 and ES-2 cells were processed for nuclear and cytoplasmic extracts. Cells were scraped, resuspended in hypotonic lysis buffer 10mM HEPES pH7.9, 10mM KCl, 0.1mM EDTA, protease inhibitors (P8849, Sigma-Aldrich) and phosphatase inhibitor cocktails 2 and 3 (P0044, P5726, Sigma-Aldrich) and incubated on ice for 20 min. After the addition of 0.25% Igepal-630 (NP40) (Sigma-Aldrich), samples were centrifuged at 3000 rpm for 5 min and supernatants containing the cytoplasmic extracts were recovered. Nuclear pellets were resuspended in 20mM HEPES pH7.9, 0.4M NaCl, 1mM EDTA with protease and phosphatase inhibitors. After three cycles of vortex and ice, samples were centrifuged at 12000 rpm for 20 min and the supernatants containing the nuclear extracts were collected.

Proteins were separated on 4–12% NuPAGE Novex Bis-Tris Protein gradient polyacrylamide gels (Thermo Fisher Scientific) and blotted onto nitrocellulose. Membranes were quenched with 5% blotto (BioRad) [42] and the proteins detected with: rabbit anti-ZNF521 (EHZF S15 sc-84808, Santa Cruz Biotechnology, DBA, Milan, Italy) at 1:1000, rabbit anti- HDAC1 (H3284, Sigma) 1:10000. Secondary rabbit HRP antibody at 1:2000 (65–6120 Thermo Fisher Scientific) was detected by the ImmunoCruz Western blotting luminal reagent (sc-2004, Santa Cruz, Biotechnology) and exposure to autoradiographic film (GE Healthcare, Milan, Italy).

### Cell proliferation assay

Transduced cells (HeyA8 and ES-2) were seeded into 6 well plates at 2.0x10^5^ cells/well in complete DMEM or RPMI respectively. Every 48 hours cells were trypsinized, counted and replated at 2.0x10^5^/well. The average of three independent cell counts, normalized to account for the dilutions performed in the individual cultures at each replating, was plotted against time to obtain a cumulative growth curve for each cell population.

The growth of HeyA8 was also evaluated using the Real-Time Glo MT Cell Viability Assay (Promega, Milan, Italy) kit, which allows the evaluation of cell proliferation based on their ATP consumption. The cells were plated in 96 wells and then the substrate and the enzyme are added in a 1:1 ratio. The plate was then incubated at 37°C and the readings (GloMax Explorer, Promega) taken at 0, 24 and 48 hours of growth.

ES-2 proliferation was also measured by MTS [3-(4,5-dimethylthiazol-2-yl)-5-(3-carboxymethoxyphenyl)-2-(4-sulfophenyl)-2H-tetrazolium, inner salt; MTS(a)] colorimetric assay. Cells were seeded at 5.0x10^2^ per well in 96-well plates in DMEM 10% FBS and assayed after 24, 48 and 72 hours of incubation. At each time point, 20μl of MTS solution were added to the wells. After 4 hours of incubation at 37°C the absorbance, which is proportional to the number of viable cells, was measured at λ = 490 nm (GloMax Explorer, Promega). All the experiments were performed in triplicate.

### Clonogenicity assay

The transduced cells are trypsinized, counted and resuspended in a medium containing DMEM F12 (GIBCO, Milan, Italy), L-glutamine (Thermo Fisher Scientific) 1%, Pen/Strep (Thermo Fisher Scientific) 1%, B27 (GIBCO) 50x, 20ng/ml hEGF (PeproTech, DBA, Milan, Italy), 2 ng/ml hFGFb (PeproTech). For ES-2 cells 10μg/ml of insulin (Sigma-Aldrich, Milan, Italy) and 4 μg/ml of heparin (Sigma-Aldrich) are added and plated in ultra-low attachment 6 wells (Corning Inc., Milan, Italy). HeyA8 were plated at a concentration of 3.0x10^4^ cells/well and ES-2 at concentration of 4.5x10^4^ cells/well. After 7 days spheres formation was observed, the cell number for every culture was calculated and the size of spheres were estimated from acquired images (at 10x magnification) by ImageJ 1.51j8. All the experiments were performed in triplicate.

### Wound healing assay–migration assay

Transduced cells were counted, seeded at the same density and cultured until >90% confluence in 6 well plates in appropriate medium with 10% FBS and then in DMEM or RPMI with reduced serum 0.5% FBS to block their proliferation. After 24 hours the monolayer of cells was scratched with a sterile 200μl pipet tip, washed with PBS 1X and incubated at 37°C in DMEM 10% FBS [43] Images were captured by phase contrast microscopy at 10x magnification, at the beginning and at regular intervals during cell migration. The migration of the cells was analysed and quantified by ImageJ 1.51j8. Experiments were performed in triplicate.

### Next generation sequencing–total RNA-Seq

RNA concentration was determined by using Quant-IT RNA Assay Kit-High Sensitivity and a Qubit Fluorometer (Life Technologies) and its quality and integrity assessed with the Agilent 4200 Tapestation System (Agilent Technologies). Total RNA–seq procedures were performed as described previously [44]. Indexed libraries were prepared using 1 μg of total RNA in technical and biological triplicate for each sample as starting material, with a TruSeq Stranded Total RNA Sample Prep Kit (Illumina Inc.). Libraries were sequenced (paired-end, 2x75 cycles) at a concentration of 8 pM/lane on NextSeq 500 platform (Illumina Inc.) [45]. Aliquots of the amplified RNA samples used for RNA-Seq remained available to be used to confirm by qRT-PCR the differential expression of selected genes.

Fastq files were aligned to the hg38 genome assembly using STAR [46]. STAR gene counts were normalized applying the median of ratios method implemented in DESeq2 R package [47]. Briefly, the normalization process implies different steps: i) for each gene, a pseudo-reference sample is created and is equal to the geometric mean across all samples; ii) for every gene in a sample and for each sample, the ratios sample/ref are calculated; iii) the median value of all ratios for a given sample is taken as the normalization factor (size factor) for that sample; iv) for each gene in each sample the normalized count values is calculated dividing each raw count value by the sample’s normalization factor.

For differential gene expression (DEG) analysis, we applied DESeq2 R package to normalized counts.

Heatmaps were generated using Cluster 3.0 for Mac OS X (C Clustering Library 1.56, Tokyo, Japan) with uncentered correlation and centroid linkage, and Java TreeView software environment (version 1.1.6r4; http://jtreeview.sourceforge.net).

Differentially expressed genes were used as input for the Molecular Signature Database Investigate gene sets analysis [48].

### Expression analysis by qRT- PCR

cDNA was synthesized using SuperScript III reverse transcriptase at 42°C and 2.5μM random hexamers (Thermo Fisher Scientific) from 1μg RNA previously prepared with Tri Reagent (Sigma-Aldrich) and verified by the NanoDrop 2000/2000c Spectrophotometer (Thermo Fisher Scientific) [49]. One cycle of 3 min at 95°C was followed by 45 cycles of 10 sec at 95°C, 10 sec at 60°C and 10 sec at 72°C, finishing with a melting curve, amplified with the iQ™ SYBR® green super mix (BioRad, Milan, Italy) using the qRT-PCR amplifier QuantStudio3 (Applied Biosystems, Milan, Italy).

Analysis of gene expression was calculated as 2^−ddCt^ and normalized for the housekeeping gene (*GAPDH*). Primers used in this study were as follows (5’-3’):

*h-ZNF521* were previously described [50];
*h-CTSK-fwd CAGGGTCAGTGTGGTTCCTG*,
*h-CTSK-rev CCCCGGTTCTTCTGCACATA*;
*h-RB1CC1 fwd TTCCACTGTTGGAGTGCCTAA*,
*h-RB1CC1 rev CATCTGCGGTATCTGGGGAC*;
*h-AIMP1-fwd ACAGCAGTAACAACCGTATCTTCTGG*,
*h-AIMP1-rev CTATTGGCTTAGAGTCGGCACTTCC*;
*h-PIBF1-fwd CTTACAAAGATTGAAGAATTGGAGG*,
*h-PIBF1-rev AATTCTTGATATTTGCTGGCATCT*;
*h-FXYD5-fwd TCCCACTGATGACACCACGA*,
*h-FXYD5 rev AAAACCAGATGGCTTGAGGGT*;
*h-HMMR-fwd ACCAACTCAAGCAACAGGAGGA*,
*h-HMMR-rev CCTGAGCTGCACCATGTTCATT*;
*h-SAMD9-fwd GGGAACTACCTTGGCTATGCAC*,
*h-SAMD9-rev CGTATTCCTGACGGTTCATTGCC*;
*h-GAPDH-fwd* CACCATCTTCCAGGAGCGAG,
*h-GAPDH-rev* TCACGCCACAGTTTCCCGGA.

Experiments were performed in triplicate.

### Statistical analysis

The student’s t-test assuming unequal variances between two samples was used to determine the significant differences (p <0,05 *, p <0,01 **, p <0,001 ***, p <0,0001 ****).

## Results

### Enforced expression of ZNF521 enhances growth of ovarian carcinoma cell lines

ZNF521 is expressed in ovary and ovarian cancers [18] and mutations of its gene (almost invariably amplifications) are present in >6% of OCs (S1A Fig) [20]. Increased expression of ZNF521 was found associated with a poor prognosis in OC patients [17–19]. Analysis of a public genomic database [51] comprising 655 OC patients confirmed an adverse prognosis for patients with ZNF521-high OCs (S1B Fig) [52].

**Fig 1:**
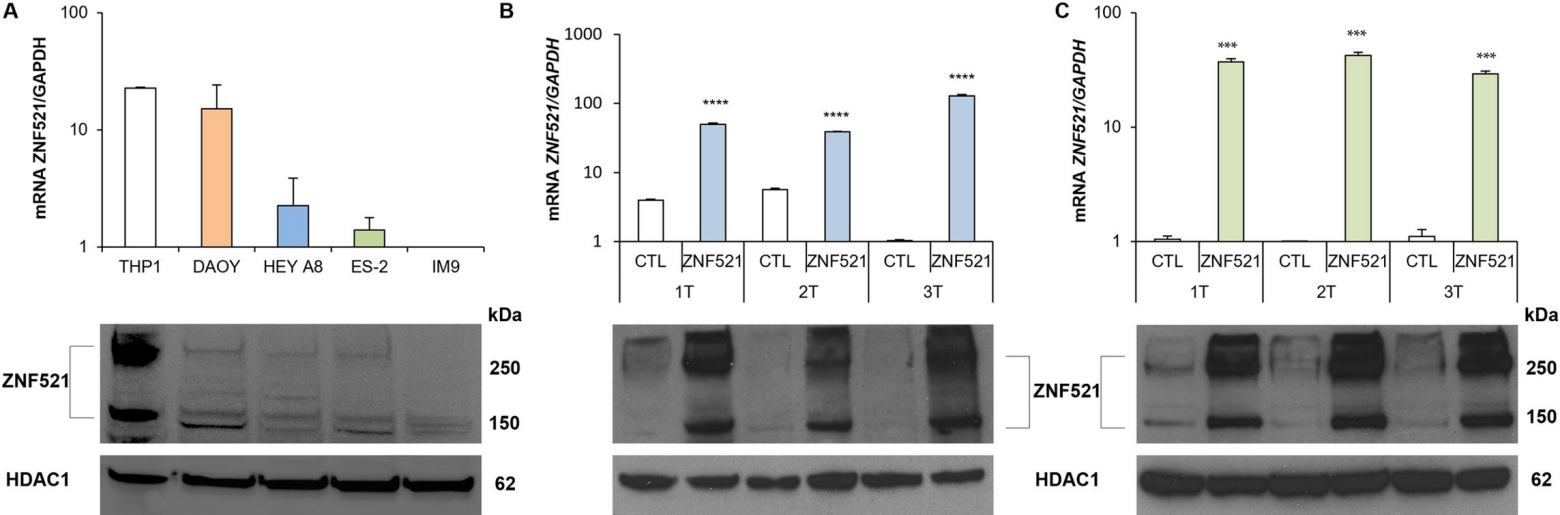
Expression of ZNF521 in HeyA8 and ES-2 human ovarian carcinoma cell lines. Endogenous ZNF521 mRNA (A, top panel: qRT-PCR) and protein (A, bottom panel: Nuclear protein detected by Western Blotting) expression in human ovarian carcinoma cell lines (HeyA8 and ES-2) compared to THP1 cells, that express high levels of ZNF521, DAOY cells, that express intermediate levels and IM9 cells that express low levels of ZNF521. Modulation of ZNF521 by lentiviral transduction mRNA (B and C, top panel: qRT-PCR) and protein expression (B and C, bottom panel: Nuclear protein detected by Western Blotting) in HeyA8 (B) and ES-2 (C) cells transduced with lentiviral vectors containing the cDNAs for ZNF521 compared to the control (CTL). “T” in 1T, 2T or 3T indicate each “Transduction”. The two bands for ZNF521 correspond to the monomer and dimers at 150kDa and 300kDa identified by the S15 anti-ZNF521 antibody. The nuclear extracts are normalized for the HDAC1 protein. Asterisks indicate p <0,05 *, p <0,01 **, p <0,001 ***, p <0,0001 ****.

To assess whether the modulation of ZNF521 expression could affect some biological characteristics of OC cells the mRNA levels of ZNF521 for HeyA8 and ES-2 OC cells were quantified by qRT-PCR and compared to that of THP1 cells, DAOY cells and of IM9 that, respectively, express very high, intermediate and very low levels of ZNF521 (Fig 1A). The HeyA8 and ES-2 cell lines, were stably transduced with two different lentiviral vectors: FUIGW (carrying only the EGFP) or FUIGW-ZNF521 (expressing the ZNF521 cDNA fused to 3xFLAG tag and EGFP) and immunoblot analysis as well as qRT-PCR confirmed a significant increase of ZNF521 expression (Fig 1B and 1C).

**Fig 2:**
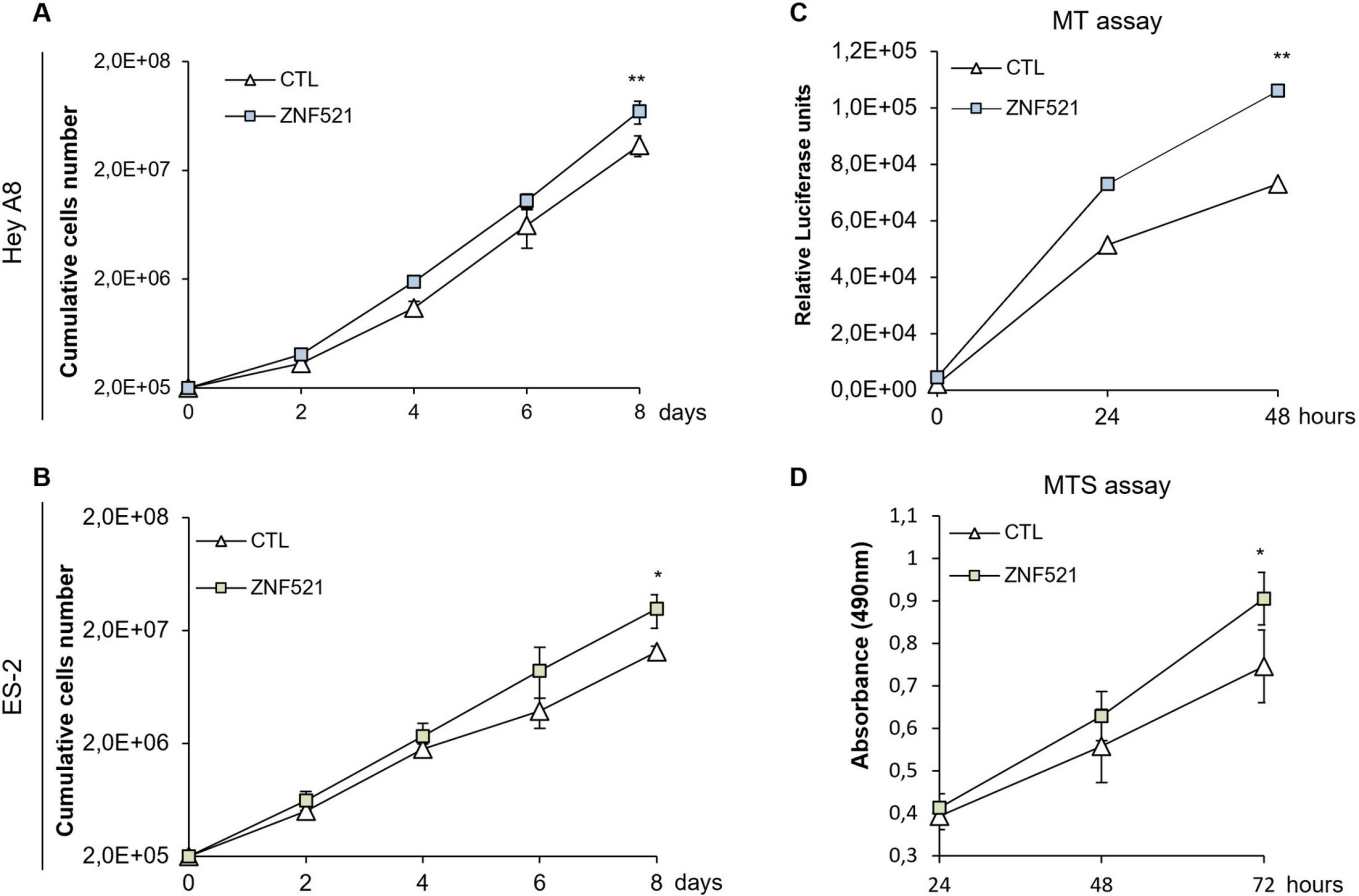
Forced ZNF521 expression confers an advantage in proliferation of OC cells. The growth of HeyA8 (A and C) and ES-2 (B and D) transduced cells was assessed by cell counts (A and B) or by MT (C) and MTS assays (D). Data shown the mean of 3 sets, each conducted in triplicate. Asterisks indicate p <0,05 *, p <0,01 **, p <0,001 ***.

Cell growth was investigated in adherent conditions and was measured by cumulative cell numbers (Fig 2A and 2B), MT (Fig 2C) and MTS assays (Fig 2D). In HeyA8 (Fig 2A and 2C), overexpression (OE) of ZNF521 induces a significant (p<0.01) increase in cell growth both at 48h (~1.5-fold increase; 1.06x10^5^ ZNF521 *vs* 7.30x10^4^ CTL cells) and after 8 days (~2-fold; 6.96x10^7^ RLU in ZNF521 *vs* 3.4x10^7^ RLU in CTL cells; MT assay) (Fig 2B and 2D). Similarly, exogenous ZNF521 expression in ES-2 increased cellular growth at 48h and up to 72 hours (~1.2-fold; 0.905 OD ZNF521 vs 0.745 OD CTL; MTS assay), and after 8 days (2.4-fold; 3.12x10^7^ ZNF521 *vs* 1.3x10^7^ CTL).

### ZNF521 improves the migration ability of human ovarian cell lines

**Fig 3:**
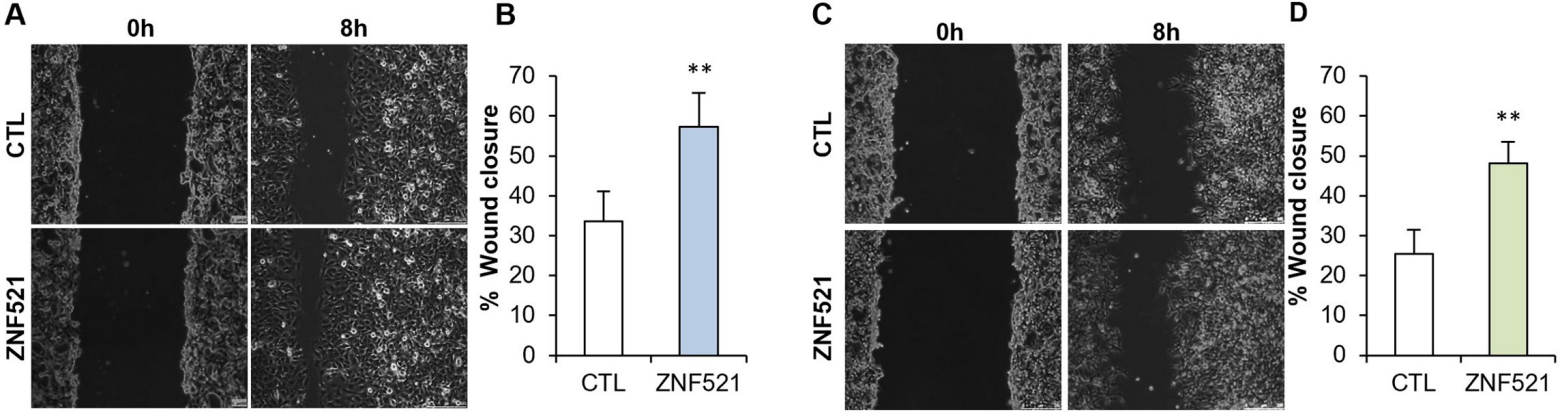
Effect of enhanced ZNF521 expression on migration ability of HeyA8 and ES-2 cell lines. ZNF521-transduced HeyA8 (A and B) and ES-2 (C and D) displayed an increased wound healing ability compared to control cells. Scale bars correspond to 250μm. As percentage of wound closure the mean of 3 sets, each conducted in triplicate, is illustrated (B and D). Asterisks indicate p <0,05 *, p <0,01 **, p <0,001 ***.

The cell migration and invasion of ZNF521-overexpressing and CTL cells was investigated by wound-healing assay. Cells were plated and grown to confluence, in 6 well plates, and then starved for 24 hours. When cell confluence approximately reached 90%, a longitudinal wound was done using a sterile tip. Cells cultures were observed at regular time intervals, monitoring the extent of the healing of the wound. Both ZNF521-overexpressing cell lines displayed a ~2-fold increase in wound healing capacity (Fig 3) compared to CTL cells.

### ZNF521 enhances clonogenicity of HeyA8 and ES-2 cells

The proliferation of HeyA8 and ES-2, ZNF521-overexpressing or CTL cells was assessed in anchorage-independent conditions to test their ability to form spheroids as readout for enrichment in CSC subpopulation in OC cell lines.

**Fig 4:**
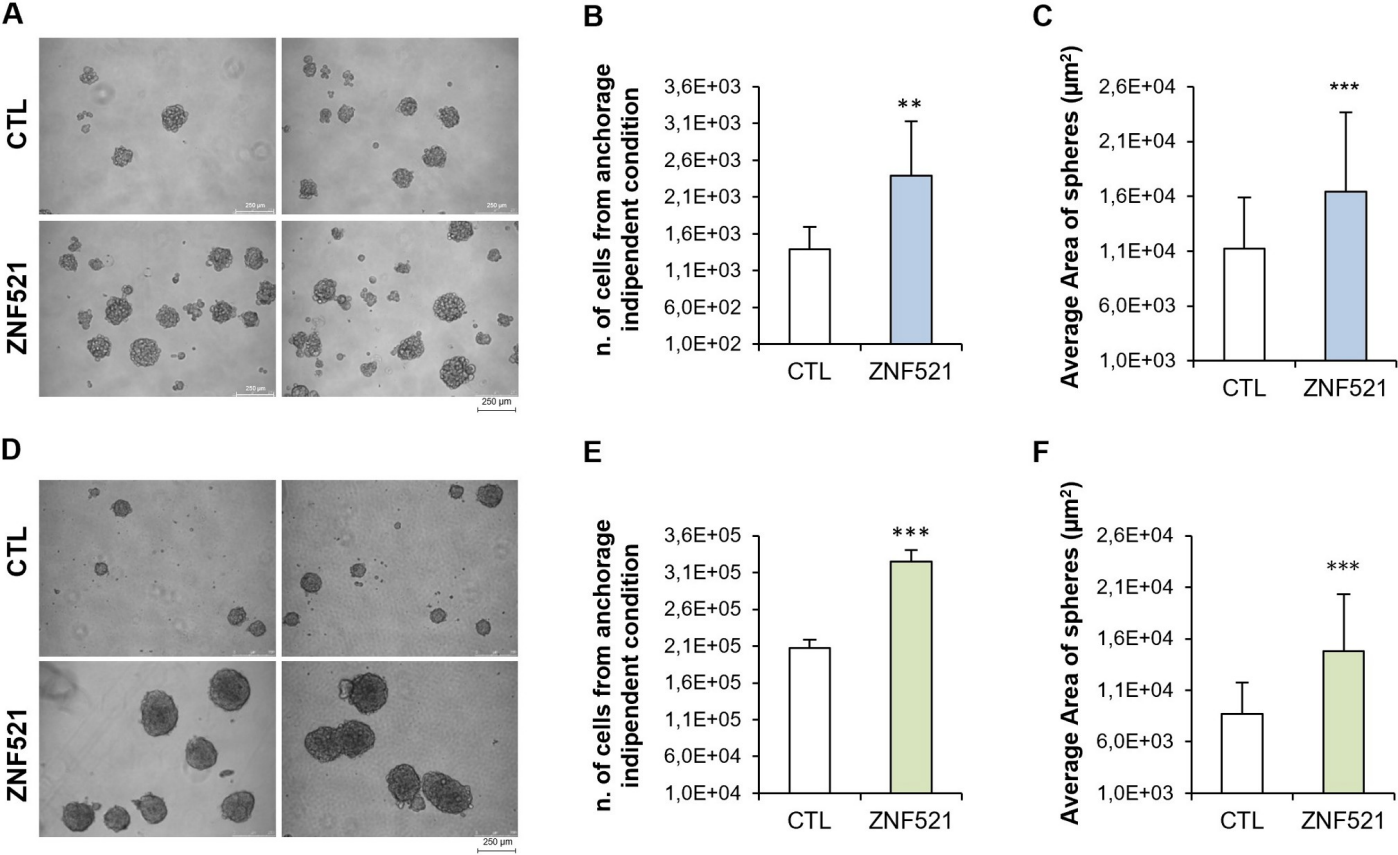
ZNF521 enforced expression enhances spheres-forming ability in HeyA8 and ES-2 cell lines. Representative images (10x) are shown for HeyA8 (A) and in ES-2 (D). Scale bars correspond to 250μm. Lentiviral-mediated enforced expression of ZNF521 in ovarian cell lines increases spheres number and size in HeyA8 (B, C) and ES-2 (E, F). Average areas of the 50 spheres are shown. The data shown here illustrate the mean of 3 sets, each conducted in triplicate. Asterisks indicate p <0,05 *, p <0,01 **, p <0,001 ***.

Overall, ZNF521-overexpressing cells showed a greater number of spheroids and of cells forming spheres (Fig 4A, 4B, 4D and 4E) compared to the controls which confirmed an enrichment in CSCs. The average areas of spheres, from images of cultures acquired by phase contrast microscopy at 10x, were calculated for 50 spheres in each culture by ImageJ 1.51j8.

Notably, ZNF521 transduced cell lines formed larger spheres than the controls (Fig 4C and 4F).

### ZNF521 over-expression reprograms the transcriptome of HeyA8 and ES-2 cells

**Fig 5:**
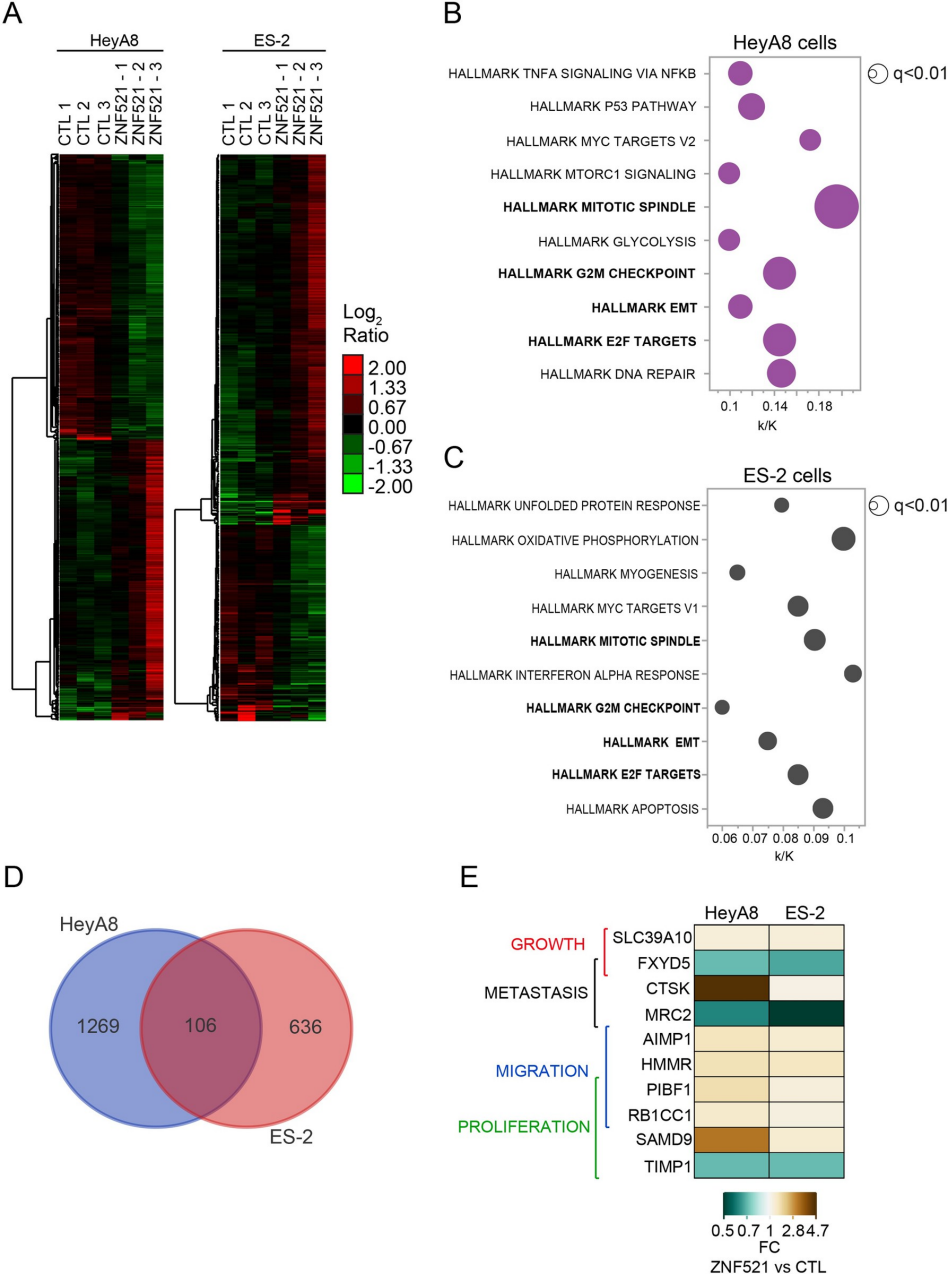
Differentially expressed genes (DEGs) by ZNF521 in hOC cell lines. (A) Hierarchical clustering of the 1375 and 742 differentially expressed genes (p<0.05) in HeyA8 and ES-2 cells, respectively. Molecular Signatures Database (MSigDB) analysis of HeyA8 (B) and ES-2 (C). (D) Size of dots represents the inverse logarithm (base 10) of statistical significance (FDR q-value) as per the legend. X-axes, fraction (k/K) of overlapping genes (k) in each Gene Set with the total number of genes represented in the specific hallmark (K). In bold, commonly enriched hallmark gene sets. Venn diagram analysis of the 1375 genes differentially expressed in HeyA8 (blu circle) and 742 genes were differentially expressed in ES-2 (red circle); 106 genes were differentially expressed in common in both cell lines. (E) Heatmap of DEGs specifically involved in growth, metastasis, migration and proliferation commonly regulated in both OC cell lines. FC, fold change (ZNF521 *vs* control).

**Fig 6:**
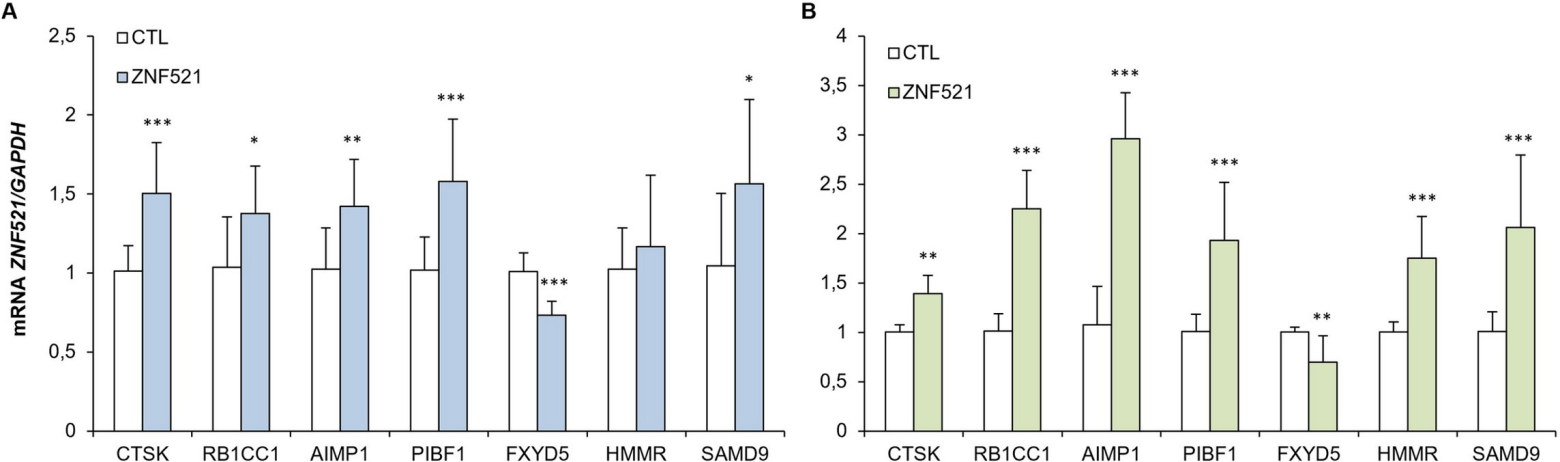
Validation of RNA-Seq data by qRT-PCR: HeyA8 (A), ES-2 (B). The data shown here illustrate the mean of three experiments each conducted in triplicate. Asterisks indicate p <0,05 *, p <0,01 **, p <0,001 ***.

Since ZNF521 is a transcription co-factor we then sought at the transcriptome of OC cell lines upon ZNF521 expression modulation. RNA-Seq analysis of both ES-2 and HeyA8 cell lines, ZNF521-OE or CTL, revealed that ZNF521 overexpression induced a large transcriptional reprogramming involving a total of 1375 and 742 differentially expressed genes (p<0.05), respectively (Fig 5A). Furthermore, Molecular Signature Database (MSigDB) focused on hallmark gene sets (see Methods) scored the enrichment of 4 hallmarks related to cancer proliferation (i.e., E2F targets, EMT, G2M checkpoint and mitotic spindle) both in HeyA8 and in ES-2 cell lines (Fig 5B and 5C), which is in line with previously described enhanced proliferation and migration of HeyA8 and ES-2 cells upon ZNF521 overexpression (Figs 2 and 3). Yet, a total of 106 genes were found overlapping between the 1375 and 742 differentially expressed genes in ZNF521-OE cells (p<0.0001, Fisher’s exact test; Fig 5D). Of these, 10 genes previously described to be associated to tumour growth, metastasis, cell migration and proliferation, were found consistently down- or up-modulated in ZNF521-OE cells *vs* CTLs (Fig 5E). Lastly, qRT-PCR analysis of 7 out of 10 genes confirmed their trend of regulation as shown in Fig 6 (A: HeyA8, B: ES-2) in both OC cell lines.

## Discussion

Ovarian carcinoma represents an important cancer-related cause of death in women, even considering that the overall survival has only modest advantage from radical debulking surgery and conventional chemotherapy [4]. The major unresolved clinical problems include malignant progression and rapid emergence of drug resistance against conventional chemotherapy. In this regard, novel strategies are warranted based on molecularly targeted therapies directed against components of specific signaling pathways required for tumour development and progression [53]. Additionally, identification of biomarkers with functional and prognostic significance, may greatly aid early diagnosis and allow timely implementation of therapeutic strategies. Profiling of 157 advanced stage serous ovarian cancers, ZNF521 (known regulator of the homeostasis in stem cells compartment and in cancer [18,22–28]) results in a set of 86-genes with significant poor overall survival profiles [17]. In exosomes derived from Bone Marrow Stromal Cells (BMSCs), ZNF521 was identified as hub protein and associated with poor outcome in gastric and ovarian cancers [18] where OCSC are shown to be implicated in the progression of ovarian carcinoma and resistance to therapy [14–16]. Moreover, recently, ZNF521 was identified as one of the top 15 genes significantly correlated with the overall survival in 1692 serous ovarian cystadenocarcinomas [19]. Taken together the analyses of different public genomic databases indicate that ZNF521 is: *i)* amplified in 6% of ovarian carcinoma cases and *ii)* associated to poor survival in ovarian cancer patients [18–20] (S1 Fig).

Here, we highlight a new role of the transcriptional co-factor ZNF521 in OC. Overall, we found that higher levels of ZNF521 in HeyA8 and ES-2 human ovarian cancer cell lines enhances their growth (Fig 2), ability to migrate (Fig 3) and to form OC spheroids (Fig 4). Such a role of ZNF521 in hOC may be exerted via the modulated expression of several regulatory genes which are relevant in cancer [54,55] (Fig 5 and S1–S3 Tables).

qRT-PCR analysis confirmed the regulation by ZNF521 of a subset of these genes by ZNF521 (N = 7; Fig 6) that, once dysregulated, induce cellular transformation and tumorigenesis.

More in depth, the data presented so far demonstrate that ZNF521 is able to regulate the expression of *HMMR* (*Hyaluronan-mediated motility receptor*) [56–60], *CTSK* (*Cathepsin K*) [61–65], *SAMD9* (*Sterile Alpha Motif Domain-Containing Protein 9*) [66,67], *AIMP1* (*Aminoacyl-tRNA synthases interacting multi-functional protein 1*) [68–71], *PIBF1* (*Progesterone-Induced Blocking Factor 1*) [72–75], *RB1CC1* (*RB1-inducible coiled-coil 1*) [76–78] and *FXYD5* [79–81]. All these genes are well known to regulate tumour progression (proliferation, migration and metastasis) by regulating EMT pathway in cancers and are also linked to poor overall survival in different types of diseases.

Human OC HeyA8 and ES-2 cell lines overexpressing ZNF521 also increase their ability to form spheroids (enrichment of CSCs). CSCs have the ability to self-renew, differentiate and possess tumorigenic properties. CSCs are also involved in drug resistance, and it has been shown that some populations of CSCs share EMT-like cellular characteristics [82]. Indeed, whole-transcriptome profiling of OC cell lines confirmed the profound transcriptional impact of ZNF521 expression modulation on a set of genes involved in tumour growth, proliferation, migration and metastasis. Forced ZNF521 expression altered the transcriptional profile of a total of 1375 and 742 genes in HeyA8 and in ES-2 human OC cell lines, respectively, as well as the enrichment of several cancer-related hallmarks including EMT regulatory genes.

Further in-depth analysis is necessary to better understand the molecular mechanism that underlines these activities of ZNF521 in human OC.

Taken together, these data are the first evidence of new role of the stem cell transcription co-factor ZNF521 (so far known for its involvement in the stem pathway) in the regulation of human epithelial ovarian carcinoma (hEOC) cells, suggesting that this could be due to the modulation exerted by ZNF521 on regulatory genes of the relevant molecular mechanisms of EMT in human ovarian cancer.

## Supporting information

S1 Fig Database analysis of ZNF521 expression in patients affected by ovarian cancer. Analysis of ZNF521 mutations in a set of 1025 patients [https://www.cbioportal.org] (A). Kaplan-Meier curve of overall survival of 655 patients [http://kmplot.com] (B).
(TIF)

S1 Table Differentially expressed genes (DEGs) in HeyA8 (A) and ES-2 (B) OC cell lines with fold change and p-value.
(XLSX)

S2 Table Hallmarks of cancer enriched in DEGs of HeyA8 (A), ES-2 (B) ZNF521 over-expressing cells, and in 106 overlapping DEGs (C).
(XLSX)

S3 Table Normalized expression counts of DEGs specifically involved in growth, metastasis, migration and proliferation in hOC cell lines analysed. (XLSX)

S1 Raw image (TIF)
