# Supplementary figures and images for: Enhanced ZNF521 expression induces an aggressive phenotype in human ovarian carcinoma cell lines
Source: PLoS One. 2022 Oct 3;17(10):e0274785. doi: 10.1371/journal.pone.0274785 (PMC9529122; doi:10.1371/journal.pone.0274785)

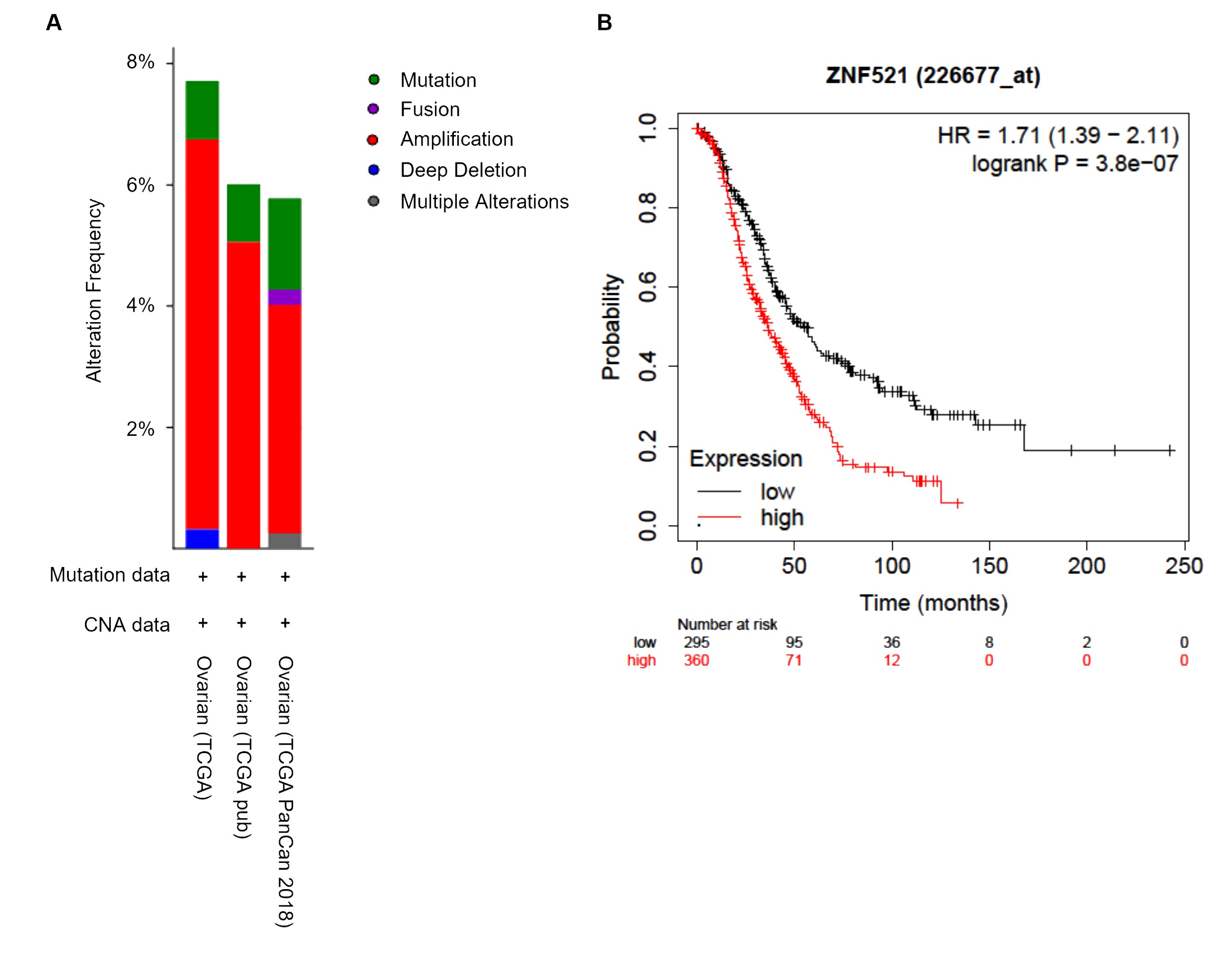

Supplement: S1 Fig — Analysis of ZNF521 mutations in a set of 1025 patients [https://www.cbioportal.org] (A). Kaplan-Meier curve of overall survival of 655 patients [http://kmplot.com] (B). (TIF) [file pone.0274785.s001.tif]

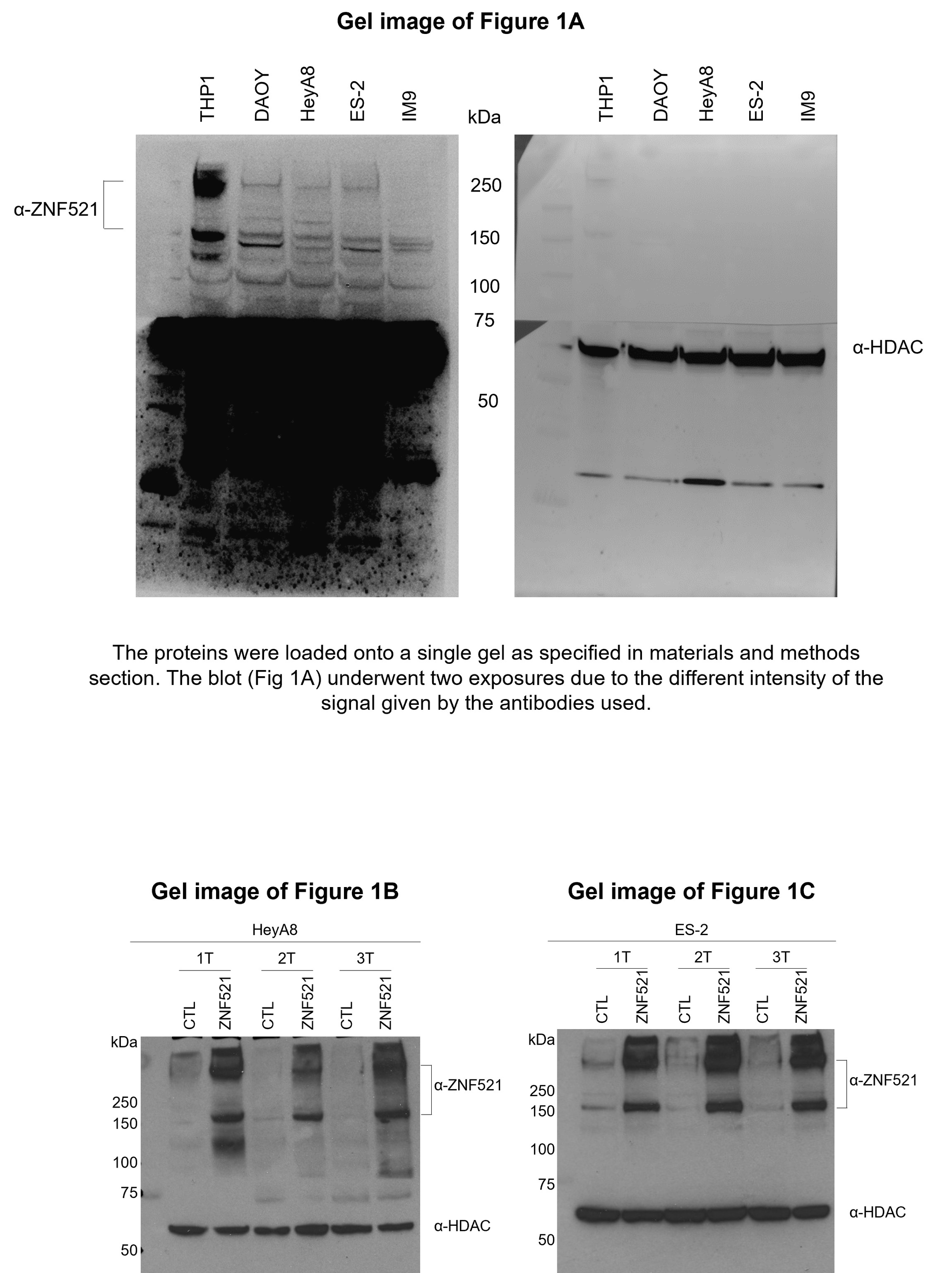

Supplement: S1 Raw image — (TIF) [file pone.0274785.s005.tif]
